# Traumatic brain injury among patients presenting from prison: a cohort study

**DOI:** 10.1038/s41598-026-37391-4

**Published:** 2026-02-02

**Authors:** Joshua Feler, Nicole Schachman, Deus Cielo, Lawrence A. Haber, Justin Berk

**Affiliations:** 1 Department of Neurosurgery, Alpert Medical School at Brown University, Providence, RI USA; 2 Johns Hopkins School of Nursing, Baltimore, MD USA; 3 Division of Hospital Medicine, Denver Health Medical Center, Denver, CO USA; 4 Department of Medicine, University of Colorado School of Medicine, Aurora, CO USA; 5 Departments of Medicine & Pediatrics, Alpert Medical School at Brown University, Providence, RI USA; 6 Center for Health and Justice Transformation, Providence, RI USA

**Keywords:** Diseases, Health care, Medical research, Neurology

## Abstract

Over two million people are incarcerated in the United States, where they experience increased illness, injury, and barriers to medical care. Traumatic brain injury (TBI) in the incarcerated population remains underexamined, including prevalence, treatment patterns, and outcomes. We analyzed the 2021–2022 National Trauma Data Bank to identify adult patients (≥ 18 years) with TBI as defined by ICD-10 codes, and incarcerated patients were identified by injury location codes. Propensity score matching (2:1 on age, sex, and race; then with the addition of Glasgow Coma Score and Injury Severity Score) was performed to balance baseline characteristics. Subgroup analysis by TBI severity (mild, moderate, severe range) was conducted. Outcomes included hospital mortality and discharge disposition; interventions received were also recorded. Patients presenting from prison with TBI were younger, more often male, and had fewer comorbidities. They presented with distinct trauma mechanisms, specifically with more frequent assaults and less frequent motor vehicle collisions and firearm injuries. After matching, there was higher mortality among patients presenting from prison with severe TBI (30.9% vs. 25.7%, *p* = 0.03), corresponding with a 43% increased risk of mortality once adjusted for age, sex, race, and Injury Severity Score despite similar treatment intensity. Discharge to skilled nursing and acute rehab was significantly less frequent among survivors presenting from prison, with absolute differences of 1.7% and 5.1%, respectively (*p* < 0.001). These findings raise concerns about equity in care delivery and post-acute services for incarcerated individuals with TBI. Structural factors impacting discharge planning in this population warrant further investigation.

**Supplementary Information:**

The online version contains supplementary material available at 10.1038/s41598-026-37391-4.

## Introduction

Over two million people are currently incarcerated in the United States^1^. The incarcerated population has an increased prevalence of medical, psychiatric, and substance use disorders relative to the non-incarcerated population, as well as an increased incidence of traumatic injury while imprisoned^2^. Patients experiencing incarceration have a constitutional right to healthcare delivery as ruled in *Estelle vs. Gamble*, a Supreme Court decision from 1973^3^, yet a variety of barriers challenge their ability to access healthcare at the community standard.

The ramifications of these obstacles may be exacerbated in trauma, especially traumatic brain injury (TBI) where delays in care and lack of access to neurosurgical services may lead to adverse outcomes^4^. TBI, the consequence of blunt and penetrating force to the head resulting in intracranial bleeding and structural brain injury, exists on a spectrum of severity. Those presenting with mild injuries may experience a concussion syndrome that improves over the course of weeks to months^5^. In severe cases, patients may require emergent neurosurgery, prolonged intensive care stays, tracheostomy, and enteral feeding tubes; even with aggressive care, patients experience high rates of mortality, functional dependence, and disorders of consciousness that may persist for many months or years^6,7^. Even patients who recover functional independence experience ongoing medical morbidity^8^.

Among patients presenting from carceral settings to a hospital for evaluation of traumatic injuries, there are varying reports about the prevalence of TBI ranging from 1% to 30% depending on setting and study type^9–11^. In one study, evaluation and management of trauma was the most common reason for presentation to the emergency room^12^ and for admission^13^ among patients experiencing incarceration. The National Trauma Data Bank has previously been explored to describe patterns of short-term outcomes after trauma occurring at prison in a study reporting increased odds of hospital mortality compared with trauma occurring in the community after adjustment for differences in baseline characteristics^14^. However, there lacks description of the subgroup of patients presenting with TBI, a group with distinct clinical characteristics and disease-specific measures of illness severity and treatment intensity. In this paper, we aim to characterize the epidemiology, management, and outcomes among patients presenting from prison with TBI.

## Methods

Cases of patients diagnosed with TBI were identified in the National Trauma Data Bank, a large convenience sample of cases reported by trauma centers across the United States, during years 2021 and 2022. The need to obtain ethical approval was waived by the Brown University Health Institutional Review board, and the need for consent was waived given the retrospective and anonymous nature of the study. All methods were carried out in accordance with relevant guidelines and regulations.

Patients presenting with TBI were identified by the ICD10 codes listed in Supplementary Table 1. Patients presenting from prison were identified by the injury location code Y92.14. Cases were included if patient age was greater than or equal to 18 years and if there was a diagnosis of TBI. Cases were excluded if missing total Glasgow Coma Score (GCS) data.

Data describing demographics, presentation, injury severity (as measured by the Injury Severity Score (ISS)), hospital characteristics, hospital events, and hospital outcomes were collected. ISS is a total body injury severity measure ranging from 0 to 75 and calculated by summing the squares of the Abbreviated Injury Scale (AIS) of the three most injured body regions. Comorbidities are documented in the database as present or absent; the list includes a total number of comorbidities calculated for each patient. The list of comorbidities included is provided in the Supplementary Methods. Craniotomy was identified by procedure codes listed in Supplementary Tables 1, and intracranial pressure monitoring was identified by presence of any type of monitoring device identified in the database.

Given the large sample size, skewness was calculated for each continuous variable to assess for asymmetry in its distribution. Following common practice, absolute skewness values greater than 1 were considered indicative of a highly skewed distribution. Highly skewed data are reported with median and interquartile range and compared with Mann-Whitney U test; otherwise, they are reported with mean and standard deviation and compared by t-test. Categorical variables were summarized with percentages and compared with chi-square testing. Counts of missing data points are reported but excluded from summative measures.

Demographics and presenting characteristics of cases were compared in the entire cohort and then in a propensity score-matched cohort on presentation from prison based on age, sex, and race at a 2:1 ratio. To assess outcomes, a second matched cohort was created after the addition of GCS and ISS to the propensity model. Major polytrauma was defined as an AIS greater than 2 in any organ system other than the head and neck. For matched cohorts, cases with missing data for age, sex, race, and GCS were excluded given the relatively small number of cases of patients presenting from prison. Subgroup analysis was performed based on GCS range defined as 3–8 for severe, 9–12 for moderate, and 13–15 for mild TBIs^15^.

Multivariable logistic regression was performed to test for association between predictors and hospital mortality, modeled with a binomial distribution and logit link. Predictors included presentation from prison, age, sex, race, body mass index, ISS, and GCS. GCS was not included in modeling for subgroup analyses in which cases were selected based on GCS range.

Analysis was performed in R (version 4.4.3). This manuscript was constructed in accordance with STROBE guidelines. The predetermined threshold for significance was a p-value < 0.05.

## Results

### Demographics and presentations of patients from prison and from the community

A total of 506,360 patients presenting from the community and 4,648 presenting from prison with TBI were identified. As summarized in Table 1, patients presenting from prison with TBI were younger on average than those from the community (mean 41.4 [SD 13.5] vs. 55.9 [SD 21.2] years, *p* < 0.001), predominantly male (95.8% vs. 64.0%, *p* < 0.001), and less frequently white (65.2% vs. 79.8%, *p* < 0.001). They had fewer comorbidities (median 1 [IQR 2] vs. 1 [IQR 3], *p* < 0.001) and a slightly lower body mass index (median 26.2 [IQR 6.4] vs. 26.3 [IQR 7.4], *p* = 0.023).

**Table 1:** Characteristics of entire prison and community groups.

	From Community (*N* = 506,360)	From Prison (*N* = 4,648)	*p* value
Age, years, mean (SD)	55.9 (21.2)	41.4 (13.5)	< 0.001
Sex			
Female	181,245 (36.0%)	194 (4.2%)	< 0.001
Male	322,720 (64.0%)	4,439 (95.8%)	
Non-Binary	132 (0.0%)	1 (0.0%)	
Missing	2,263	14	
Race			
White	366,811 (79.8%)	2,708 (65.2%)	< 0.001
Asian	13,767 (3.0%)	40 (1.0%)	
Black	65,574 (14.3%)	1,265 (30.4%)	
Pacific Islander	1,509 (0.3%)	17 (0.4%)	
Unknown	11,967 (2.6%)	125 (3.0%)	
Missing	46,732	493	
Body Mass Index, median (IQR)	26.3 (7.4)	26.2 (6.4)	
Missing	57,347	554	0.023
Total Comorbidities, median (IQR)	1 (3)	1 (2)	< 0.001
Hospital Type			
For-profit	62,427 (12.3%)	484 (10.4%)	< 0.001
Government	4,480 (0.9%)	85 (1.8%)	
Non-profit	439,164 (86.8%)	4,073 (87.7%)	
Missing	289	6	
Bedsize			
≤200	36,837 (7.3%)	275 (5.9%)	< 0.001
201–400	144,807 (28.6%)	1,119 (24.1%)	
401–600	147,267 (29.1%)	1,493 (32.1%)	
>600	177,449 (35.0%)	1,761 (37.9%)	
Hospital Trauma Level			
I	232,919 (60.0%)	2,757 (73.0%)	< 0.001
II	132,028 (34.0%)	831 (22.0%)	
III	23,466 (6.0%)	191 (5.1%)	
Missing	117,947	869	
ISS, mean (sd)	12 (13)	9 (11)	< 0.001
Missing	294	2	
Presenting GCS, median (IQR)	15 (1)	15 (1)	0.815
Missing	20,619	229	
GCS Category			
Mild	375,315 (77.3%)	3,544 (80.2%)	0.001
Moderate	40,096 (8.3%)	318 (7.2%)	
Severe	70,330 (14.5%)	557 (12.6%)	
Severe Polytrauma	113,822 (22.5%)	467 (10.0%)	< 0.001

IQR: interquartile range; SD: standard deviation; ISS: injury severity score; GCS: Glasgow coma score;

Injury severity, as measured by ISS, was lower in the prison group (median 9 [IQR 11] vs. 12 [IQR 13], *p* < 0.001). Patients from prison were more often treated at larger hospitals with more than 400 beds (70.0% vs. 64.1%, *p* < 0.001), which were less frequently for-profit (10.4% vs. 12.3%, *p* < 0.001), and more often had level I trauma certification (73.0% vs. 60.0%, *p* < 0.001). Presenting GCS was statistically higher among the prison group (median 15 [IQR 1] v. 15 [IQR 1], *p* < 0.001), a difference better reflected in the lesser representation of moderate (7.2% vs. 8.3%, *p* < 0.001) and severe (12.6% vs. 14.5%, *p* < 0.001) range injures. Major polytrauma was less common in the prison group (10.0% vs. 22.5%, *p* < 0.001).

Due to removal of cases with missing covariates in the match model, 723 (15.5%) were excluded from prison group, and 60,087 (13.4%) were excluded from the community group entering the matching process. Once matched for age, sex, and race, there were 3,925 in the prison group and 7,850 in the community group in the first matched cohort. There were no differences in these demographic covariates (Supplementary Table 2), but there remained significant differences in trauma severity. Total comorbidity counts were similar (overall median 1 [IQR 2], *p* = 0.45), while BMI was lower among patients from prison (median 26.0 [IQR 6.3] vs. 26.5 [IQR 7.2], *p* < 0.001).

As shown in Table 2, the prison group had a lower mean ISS compared to the community group (median 10 (IQR 11) vs. 14 (IQR 13), *p* < 0.001) and a higher mean GCS score (median 15 (IQR 1) vs. 15 (IQR 3), *p* < 0.001); within it, there were fewer patients in the severe TBI GCS category (12.9% vs. 20.9%), and with major polytrauma (10.3% vs. 28.8%, *p* < 0.001). Injury patterns differed substantially: penetrating trauma was less common in the prison group (6.1% vs. 8.2%, *p* < 0.001), while blunt trauma predominated (93.9% vs. 91.8%, *p* < 0.001). Trauma intent differed, with assault accounting for the majority of prison cases (62.6% vs. 15.0%, *p* < 0.001), compared to mostly unintentional injuries in the community group (33.2% vs. 81.5%, *p* < 0.001). Self-inflicted injuries were slightly more frequent in the prison group (3.0% vs. 2.6%, *p* < 0.001). Mechanisms of injury also varied: the prison group was far less frequently involved in motor vehicle trauma—including occupants, pedestrians, and motorcyclists (0.2% vs. 45.4%, *p* < 0.001)—and firearm injuries (0.1% vs. 7.2%, *p* < 0.001).

**Table 2:** Injury information age-, sex-, and race-matched cohorts.

	From Community (*N* = 7,850)	From Prison (*N* = 3,925)	*p* value
ISS, median (IQR)	14 (13)	10 (11)	< 0.001
Presenting GCS, median (IQR)	15 (3)	15 (1)	< 0.001
GCS Category			
Mild	5,506 (70.1%)	3,142 (80.1%)	< 0.001
Moderate	702 (8.9%)	278 (7.1%)	
Severe	1,642 (20.9%)	505 (12.9%)	
Severe Polytrauma	2,257 (28.8%)	404 (10.3%)	< 0.001
Trauma Intent			
Assault	1,148 (15.0%)	2,205 (62.6%)	< 0.001
Other	21 (0.3%)	31 (0.9%)	
Self-inflicted	200 (2.6%)	106 (3.0%)	
Undetermined	46 (0.6%)	14 (0.4%)	
Unintentional	6,220 (81.5%)	1,169 (33.2%)	
N-Miss	215	400	
Trauma mechanism			
Fall	2,012 (26.4%)	1,194 (33.9%)	< 0.001
MVT-Associated Trauma	3,465 (45.4%)	6 (0.2%)	
Struck By/Against	944 (12.4%)	2,095 (59.4%)	
Other	1,429 (18.2%)	630 (16.1%)	
N-Miss	223	400	
Trauma type			
Blunt	7,003 (91.8%)	3,311 (93.9%)	< 0.001
Penetrating	623 (8.2%)	214 (6.1%)	
N-Miss	224	400	

ISS: injury severity score; IQR: interquartile range; GCS: Glasgow coma score; MVT: motor vehicle transport.

After the addition of GCS and ISS to the propensity score, there were 3,925 patients in the prison group and 7,850 in the community group (Table 3). The matched covariates were not different between the groups (Supplementary Table 3). Comorbidity count remained similar between groups (median 1 [IQR 2], *p* = 0.37), while BMI remained minimally lower in the prison group (median 26.0 [IQR 6.3] vs. 26.5 [IQR 7.2], *p* < 0.001).

**Table 3:** Treatments and outcomes of the age-, sex-, race-, GCS-, and ISS-matched cohort.

	From community (*N* = 7,850)	From Prison (*N* = 3,925)	*p* value
Intracranial pressure monitoring	182 (2.3%)	105 (2.7%)	0.237
Craniotomy	254 (3.2%)	153 (3.9%)	0.064
Hospital discharge disposition			
Against Medical Advice	329 (5.2%)	53 (1.7%)	< 0.001
ARF	464 (7.3%)	70 (2.2%)	
Deceased	306 (4.8%)	195 (6.2%)	
Home	4,415 (69.5%)	1,704 (54.5%)	
Home with Services	304 (4.8%)	20 (0.6%)	
Hospice	45 (0.7%)	11 (0.4%)	
Law Enforcement	56 (0.9%)	881 (28.2%)	
SNF	213 (3.4%)	53 (1.7%)	
Other	217 (2.8%)	138 (3.5%)	
N-Miss	1,500	800	
Intensive Care LOS, days, median (IQR)	3 (4)	3 (4)	0.001
Hospital LOS, days, median (IQR)	3 (5)	3 (4)	0.45
Hospital Mortality	306 (3.9%)	195 (5.0%)	0.007

IQR: interquartile range; ARF: acute rehab facility; SNF: skilled nursing facility; LOS: length of stay.

### Treatments and outcomes

Among this GCS- and ISS-matched cohort, patients in the prison group more frequently transferred to another hospital (5.2% vs. 3.2%, *p* < 0.001). Use of intracranial pressure monitoring (2.7% vs. 2.3%, *p* = 0.237) and craniotomy/craniectomy (3.9% vs. 3.2%, *p* = 0.064) were not different between groups. Hospital discharge disposition varied, with decreased rates of discharge against medical advice (1.7% vs. 5.2%, *p* < 0.001), to acute rehabilitation facilities (ARF) (2.2% vs. 7.3%, *p* < 0.001) and to skilled nursing facilities (SNF) (1.7% vs. 3.4%, *p* < 0.001) in the prison group. This gap was more pronounced in patients over 65 years old (ARF 3.9% vs. 13.9%, SNF 3.9% vs. 14.9%, *p* < 0.001). Total hospital length of stay (LOS) was similar between groups (median 3 [IQR 4], *p* = 0.45).

In this same cohort, hospital mortality was more frequent among the prison group (5.0% vs. 3.9%, *p* = 0.007). Among patients with severe TBI, patients from prison exhibited significantly higher mortality compared to patients from the community (30.9% vs. 25.7%, *p* = 0.03). Once adjusted for age, sex, race, and ISS, presentation from prison was associated with a 43% increased risk of hospital mortality among those with severe traumatic brain injury (aOR 1.43, 95%CI [1.10, 1.86], *p* = 0.008). The odds of mortality did not vary between the mild (aOR 1.1, 95%CI [0.63, 1.81], *p* = 0.8) and moderate GCS ranges (aOR 1.77, 95%CI [0.87, 3.59], *p* = 0.11) based on presentation from prison (Supplementary Table 4).

## Discussion

We found that TBI among patients presenting from prison is an epidemiologically distinct entity from TBI among patients presenting from the community. When matched for age, sex, and race, measures of injury severity were lower among the group from prison, and mechanisms of injury were demonstrably different. Once matched for injury severity, presentation from prison was independently associated with increased risk of hospital mortality among patients presenting with TBI, an effect driven by a 43% increased risk of mortality for patients presenting from prison in the severe TBI GCS range.

Among all patients studied, those from prison were younger, more often Black, and more often male, reflecting underlying differences between the incarcerated and non-incarcerated populations^1^. Perhaps because of limited access to firearms and motor vehicles, related trauma involving their use was minimally represented among patients from prison. We hypothesize that the absence of these high-energy trauma mechanisms may have contributed to overall lower severity of injury among the prison group in the age-, race-, and sex-matched cohorts. The relative preponderance of self-harm as the intent of injury was also notable and is consistent with the findings of a prospectively collected multicenter cohort study in which self-injurious behavior resulted in nearly a fifth of trauma activations among incarcerated patients studied^11^. Patients experiencing incarceration are at elevated risk for suicidality, and it is a frequent cause of death within this group^16,17^.

Prior research regarding mortality after brain trauma for patients experiencing incarceration is sparse. In our study, we observed an increased frequency of hospital mortality among all TBI grades and specifically a 43% increase in adjusted odds of death associated with presentation from prison among patients with severe TBI. Shahrestani et al. reported on patients presenting with traumatic acute subdural hematoma, a subgroup of patients presenting with TBI, in the National Inpatient Sample and discovered no difference in hospital mortality based on presentation from prison; however, there was a higher proportion of patients with severe TBI in the community group,^18^ so differences of baseline risk might have obscured the effects of presentation from prison. Similarly, a study of patients with diverse trauma types in the 2015–2016 TQIP database did not identify a significant difference in mortality between patients presenting from prison and from the community before or after adjustment for confounders that included GCS,^10^ though they did not perform propensity score matching.

The causes underlying the difference in mortality seen in our study warrant further investigation. Differences in injury mechanism were not modeled and could impact outcomes through variable patterns of injury with dissimilar contributions to mortality risk despite similar ISS. While it was expected that the relative absence of the high energy motor vehicle trauma and firearm-related injuries among patients presenting from prison would confer a protective effect against mortality in this group,^19^ this effect was not observed or may have been countervailed by a competing harmful effect associated with presentation from prison.

While there was a preponderance of assault as a mechanism of injury among patients presenting from prison, its effect on the risk of mortality requires further study in this population. Prior studies of violence as a risk factor for short-term mortality after trauma have focused on injuries in the community. One study identified that intentional TBI produced statistically similar albeit numerically lower measures of injury severity and odds of hospital mortality^20^. Another study including all trauma types reported an increased risk of hospital mortality among those injured intentionally but was contaminated by high prevalence of firearm-related violence, limiting transferability to the prison population^21^.

Our study assessed the total, rather than direct, effects of presentation from prison, which may include effects mediated through differences in clinical course after arrival to the hospital. Although the rates of measurable TBI-specific interventions—craniotomy or ICP monitoring—did not differ significantly between groups, these metrics are limited proxies for the quality of care. The similarity in utilization does not preclude differences in timeliness or appropriateness of interventions that may be delayed by complexities in decision-making for incapacitated patients, collaboration with Department of Corrections staff, or other structural differences that alter workflows^2^. For example, the observation of increased hospital transfer rate raises the question of under-triage among patients presenting from prison, which could contribute to delays in definitive care. The hypothesis that unmeasured heterogeneity in clinical care contributes to worsened outcomes among the patients presenting from prison might additionally be supported by the finding of increased risk for mortality seen only among patients with severe TBI, fragile patients whose outcomes are may be more sensitive to difference in care quality and intensity. Future study should investigate variation in otherwise routine clinical care to evaluate for these effects.

Finally, discharge disposition varied greatly and meaningfully among TBI survivors with fewer patients from prison discharged to inpatient acute rehabilitation and skilled nursing facilities. Lacking data on neurological state at discharge, it is impossible to conclude that these services were as needful among patients experiencing incarceration, however it is possible that these patients had limited access to post-acute care services based on their incarceration status^22^. Facilities may consider incarceration status in evaluating a patient for admission, the disclosure of which may lead to decreased odds of acceptance, as was shown in a study of SNF acceptances of patients approved for compassionate release from prison in the setting of severe illness^23^. Additionally, patients discharged to an inpatient facility must remain accompanied by correctional officers, creating disincentive for prison systems to support this practice. Alternatively, it is possible that disposition to a post-acute care facility was less needed because some prisons and jails have on-site medical services including physical and occupational therapy routing discharging patients back to carceral facilities.

### Implications of findings

Beyond the need to identify and address contributors to increased mortality among patients presenting from prison with severe traumatic brain injuries, the size of this cohort – nearly 4,000 in a single year from this convenience sample that does not capture the entire national burden of the disease process – highlights the urgent need for clear procedures for management of patients presenting with brain injury while incarcerated. This is a population who must be managed through a specific ethical, legal, and logistical framework both while hospitalized and after discharge^13,24^ that may be unfamiliar to hospital staff.

Many of these patients, especially those with moderate and severe GCS range injuries, may lose decisional capacity at least transiently and require complex medical decision-making that depends heavily on individual patient preferences for and beliefs about the acceptability of treatments and specific long-term neurological outcomes. Even with an identified medical surrogate, communication may be restricted and mediated by the correctional system to the extreme of barring all visitors and communication with friends and family if deemed necessary to protect the public interest. In practice, the process of medical decision-making for incapacitated patients has been observed to be both variable and imperfectly performed^24,25^.

Post-hospital care is also a point of special consideration. As mentioned, there are rehabilitative resources at some prison facilities to support the recovery of patients discharging from a hospital that might divert patients from typical post-acute care facilities. Yet, these prison resources may be limited and designed with different therapeutic intent than providing rehabilitative care to patients with recent brain injuries, which could contribute to divergent long-term outcomes. Especially among the growing population of older adults who are incarcerated, this places a large financial and logistical burden^26,27^ on prison health resources. Recovery of function after TBI occurs over years across the spectrum from mild^5^ to severe^7^ injuries. Differential access to relevant services through this period might potentiate differential long-term outcomes.

### Strengths and limitations

Strengths of this study include its large sample size drawn from many institutions, use of propensity score matching to address covariate imbalance, and stratification by severity of TBI in assessment outcomes.

Registry-based research about health outcomes among carceral populations remains a nascent field. Although injury location codes are theoretically sound with an overtly reasonable definition, their sensitivity and specificity remain to be characterized. It was assumed that patients identified as injured at prison were incarcerated, though some, presumed small, number may instead have been injured while working at or visiting a prison. Moreover, small and unique clinical populations may challenge coding conventions designed for the general population. For example, it seems improbable that approximately half of the patients from prison were discharged “home” unless “home” referred to a carceral facility which might otherwise have been coded as discharge to “law enforcement.” These limitations present opportunities for further study to elucidate the characterization of patients who are incarcerated in large registries.

Approximately 13% of all cases were excluded due to missingness of key data points, though this proportion was similar between the groups. Hospital mortality and disposition are narrow measures of outcome after TBI as most patients survive and experience recovery after hospital discharge, leaving the possibility of further divergence of outcomes based on incarceration status. The findings presented here are specific to patients presenting from prisons in the United States and may not be transferrable to patients presenting from prisons in other nations. Heterogeneity in justice systems and carceral medicine programs may precipitate measurable differences in demographics, trauma mechanisms, and clinical outcomes. Nonetheless, within the context of hypothesis-generating research, these limitations might be tolerated while awaiting confirmatory studies.

## Conclusions

This study identifies a significant disparity in hospital mortality among incarcerated patients with isolated severe TBI despite similar treatment intensity after propensity score matching for baseline differences in demographics. Furthermore, disparities in discharge disposition—including lower rates of rehabilitation referrals—suggest the possibility of inequitable post-hospital care. Further research is required to elucidate the underlying causes of this disparity. Future work might investigate variation in care quality that is unmeasured in national registry datasets, differences in medical decision-making processes and outcomes, and broader structural differences in the longitudinal evaluation and management of traumatically injured patients presenting from carceral facilities.

## Supplementary Information

Below is the link to the electronic supplementary material.

Supplementary Material 1

Supplementary Material 2

## Data Availability

Study data is available upon reasonable request. Contact the corresponding author for further information.

## Appendix

Binary Comorbidity Data Included from Data Set

- Alcohol use disorder
- Angina pectoris
- Bleeding disorder
- Cerebral vascular accident
- Chronic obstructive pulmonary disease
- Chronic renal failure
- Cirrhosis
- Congenital anomalies
- Congestive heart failure
- Current smoker
- Currently receiving chemotherapy for cancer
- Dementia
- Diabetes mellitus
- Disseminated cancer
- Functionally dependent health status
- Hypertension
- Myocardial infarction
- Peripheral arterial disease
- Steroid use
- Substance use disorder
