# Supplementary material for: Traumatic brain injury among patients presenting from prison: a cohort study
Source: Sci Rep. 2026 Feb 2;16:13388. doi: 10.1038/s41598-026-37391-4 (PMC13109346; doi:10.1038/s41598-026-37391-4)
Supplement: Supplementary file 1 — Supplementary Material 1 [file 41598_2026_37391_MOESM1_ESM.docx]

**Supplementary Table 1:** ICD-10 code definitions

| Category | Codes |
| --- | --- |
| Traumatic Brain Injury | S02.0, S02.1, S02.8, S02.91, S04.02, S04.03, S04.04, S06, S07.1 |
| Craniotomy | 00933ZZ, 00940ZZ, 00C00ZZ, 00C10ZZ, 00C20ZZ, 00C40ZZ, 00N00ZZ, 00N20ZZ, 00N70ZZ, 009440Z, 00943ZZ, 009400Z, 009430Z, 00940ZZ, 00944ZZ, 009330Z, 00934ZZ, 009340Z, 00930ZZ, 009300Z |

**Supplementary Table 2:** Summary of age-, sex-, and race-matched cohort before and after matching

| **Variable** | **Treated (Before)** | **Control (Before)** | **Std. Diff (Before)** | **Treated (After)** | **Control (After)** | **Std. Diff (After)** |
| --- | --- | --- | --- | --- | --- | --- |
| **AgeYears** | 41.606 | 56.666 | -1.124 | 41.606 | 41.606 | 0.000 |
| **distance** | 0.018 | 0.009 | 0.953 | 0.018 | 0.018 | 0.000 |
| **raceAsian** | 0.010 | 0.030 | -0.202 | 0.010 | 0.010 | 0.000 |
| **raceBlack** | 0.304 | 0.143 | 0.349 | 0.304 | 0.304 | 0.000 |
| **racePacific Islander** | 0.004 | 0.003 | 0.009 | 0.004 | 0.004 | 0.000 |
| **raceUnknown** | 0.031 | 0.026 | 0.027 | 0.031 | 0.031 | 0.000 |
| **raceWhite** | 0.652 | 0.797 | -0.305 | 0.652 | 0.652 | 0.000 |
| **SEXF** | 0.042 | 0.366 | -1.615 | 0.042 | 0.042 | 0.000 |
| **SEXM** | 0.958 | 0.634 | 1.611 | 0.958 | 0.958 | 0.000 |
| **SEXNB** | 0.000 | 0.000 | 0.000 | 0.000 | 0.000 | 0.000 |

**Supplementary Table 3:** Summary of age-, sex-, race-, GCS-, and ISS-matched cohort before and after matching

| **Variable** | **Treated (Before)** | **Control (Before)** | **Std. Diff (Before)** | **Treated (After)** | **Control (After)** | **Std. Diff (After)** |
| --- | --- | --- | --- | --- | --- | --- |
| **AgeYears** | 41.61 | 56.67 | -1.12 | 41.61 | 41.38 | 0.02 |
| **distance** | 0.02 | 0.01 | 0.90 | 0.02 | 0.02 | 0.00 |
| **ISS** | 11.76 | 14.88 | -0.38 | 11.76 | 11.93 | -0.02 |
| **raceAsian** | 0.01 | 0.03 | -0.20 | 0.01 | 0.01 | 0.00 |
| **raceBlack** | 0.30 | 0.14 | 0.35 | 0.30 | 0.31 | -0.01 |
| **racePacific Islander** | 0.00 | 0.00 | 0.01 | 0.00 | 0.00 | 0.02 |
| **raceUnknown** | 0.03 | 0.03 | 0.03 | 0.03 | 0.03 | 0.01 |
| **raceWhite** | 0.65 | 0.80 | -0.31 | 0.65 | 0.65 | 0.00 |
| **SEXF** | 0.04 | 0.37 | -1.62 | 0.04 | 0.04 | -0.01 |
| **SEXM** | 0.96 | 0.63 | 1.61 | 0.96 | 0.96 | 0.00 |
| **SEXNB** | 0.00 | 0.00 | 0.00 | 0.00 | 0.00 | 0.01 |
| **TOTALGCS** | 13.26 | 13.00 | 0.07 | 13.26 | 13.37 | -0.03 |

**Supplementary Table 4:** Adjusted odds ratios of multivariable regression for hospital mortality among mild, moderate, and severe TBI ranges

| Characteristic | Mild | | | Moderate | | | Severe | | |
| --- | --- | --- | --- | --- | --- | --- | --- | --- | --- |
|  | **aOR** | **95% CI** | **p-value** | **aOR** | **95% CI** | **p-value** | **aOR** | **95% CI** | **p-value** |
| Presenting from Prison | 1.08 | 0.63, 1.81 | 0.8 | 1.77 | 0.87, 3.59 | 0.11 | 1.43 | 1.10, 1.86 | **0.008** |
| Age, years | 1.06 | 1.05, 1.08 | **<0.001** | 1.05 | 1.03, 1.08 | **<0.001** | 1.02 | 1.01, 1.03 | **<0.001** |
| Race |  |  |  |  |  |  |  |  |  |
| White | — | — |  | — | — |  | — | — |  |
| Asian | 2.22 | 0.33, 8.27 | 0.3 | 0.00 |  | >0.9 | 1.90 | 0.41, 8.88 | 0.4 |
| Black | 0.99 | 0.53, 1.74 | >0.9 | 1.33 | 0.61, 2.79 | 0.5 | 0.61 | 0.43, 0.84 | **0.003** |
| Pacific Islander | 0.00 | 0.00, 419,790,453,244 | >0.9 | 0.00 |  | >0.9 | 1.52 | 0.19, 9.64 | 0.7 |
| Unknown | 1.77 | 0.42, 5.10 | 0.4 | 0.33 | 0.01, 2.26 | 0.4 | 1.44 | 0.85, 2.38 | 0.2 |
| SEX |  |  |  |  |  |  |  |  |  |
| F | — | — |  | — | — |  | — | — |  |
| M | 0.60 | 0.24, 2.03 | 0.3 | 2.18 | 0.41, 40.7 | 0.5 | 1.0 | 0.55, 1.85 | >0.9 |
| ISS | 1.11 | 1.08, 1.13 | **<0.001** | 1.10 | 1.06, 1.14 | **<0.001** | 1.09 | 1.08, 1.11 | **<0.001** |
| Abbreviations: CI = Confidence Interval, aOR = Adjusted Odds Ratio | | | | | | | | | |
