# Supplementary material for: Traumatic brain injury among patients presenting from prison: a cohort study
Source: Sci Rep. 2026 Feb 2;16:13388. doi: 10.1038/s41598-026-37391-4 (PMC13109346; doi:10.1038/s41598-026-37391-4)
Supplement: Supplementary file 2 — Supplementary Material 2 [file 41598_2026_37391_MOESM2_ESM.pdf]

Lifespan
Delivering health with care.℠

Research Protection Office
Office of Research
Coro East, Suite 1A, Room 130
167 Point Street
Providence, RI 02903-4771
Tel 401 444-6246, Fax 401 444-7960

E. P. Bradley Hospital
Rhode Island Hospital
The Miriam Hospital
Newport Hospital
Gateway Healthcare

September 16, 2025

TO:	Deus Cielo, MD
FROM:	Research Protection Office
SUBJECT:	IRB Determination: RESEARCH - NOT HSR

PROJECT TITLE:	[2352752-1] Hospital outcomes after traumatic brain injury in a national registry
CMTT/PROJ:	411725

DETERMINATION:	RESEARCH - NOT HSR
EFFECTIVE DATE:	September 15, 2025

This package has received Administrative Review based on applicable federal regulations and institutional policy. The Lifespan IRB 2 reviewed the New Project package as part of the above referenced project title in accordance with 45 CFR 46 and determined this project is RESEARCH - NOT HSR.

Based upon the information provided in this package:

The research is NOT RESEARCH INVOLVING HUMAN SUBJECTS; 45 CFR 46 does not apply.
Human subject means a living individual about whom an investigator (whether professional or student) conducting research:

- Obtains information or biospecimens through intervention or interaction with the individual, and uses, studies, or analyzes the information or biospecimens; or
- Obtains, uses, studies, analyzes, or generates identifiable private information or identifiable biospecimens.

An Activity Report is due by September 14, 2027 to confirm if the activity is ongoing or complete.
The Activity Report is due 60 days before the Next Report Due date.

Lifespan Research Data Policy
Any research data that includes Protected Health Information (PHI) or a Limited Data Set (LDS), as defined by HIPAA Regulations, may only be stored on:

1. Lifespan managed storage platforms that comply with Lifespan policy "HSP-86.1 Data Backup and Storage Policy";
2. Lifespan managed computer workstations that comply with policy "HSP-90 Workstation Use Policy"; and
3. Mobile devices that comply with "HSP-102 Mobile Device Management Policy".

Generated on IRBNet

This includes data that originates from a Lifespan affiliated Covered Entity, personally identifiable information of Lifespan employees, or data originating from Lifespan or its affiliates that is classified as confidential.

For more information contact Lifespan IT department or Director, Research Protection Office.

IRB Compliance
The Lifespan IRB 2 complies with HHS 45 CFR 46, FDA 21 CFR Parts 50 and 56 and other federal and state laws and regulations, as applicable, as well as ICH-GCP as they correspond to the FDA/DHHS regulations.

Federalwide Assurance (FWA)

Rhode Island Hospital (RIH): FWA00001230
The Miriam Hospital (TMH): FWA00003538
Emma Pendleton Bradley: FWA00001129
Newport Hospital: FWA00003435
Gateway Healthcare: FWA00022347

OHRP IRB Registration

RIH IRB 1: IRB00000396
RIH IRB 2: IRB00004624
TMH IRB: IRB00000482

The following items are acknowledged in this submission:

- Application Form - FORM_HSR Determination 06102020.docx (UPDATED: 08/14/2025)
- CV/Resume - Cielo-CV.pdf (UPDATED: 08/14/2025)
- Data Collection - example_data_fields.xlsx (UPDATED: 08/14/2025)
- Lifespan - Basic Information Form - Lifespan - Basic Information Form (UPDATED: 08/29/2025)
- Protocol - ProtocolV1_1.docx (UPDATED: 08/14/2025)

This document has been electronically signed in accordance with all applicable regulations, and a copy is retained within our records.

Generated on IRBNet
